# A Ratiometric Biosensor Containing Manganese Dioxide Nanosheets and Nitrogen-Doped Quantum Dots for 2,4-Dichlorophenoxyacetic Acid Monitoring

**DOI:** 10.3390/bios14020063

**Published:** 2024-01-24

**Authors:** Yang Guo, Jingran Sun, Mingzhu Liu, Jin Wu, Zunquan Zhao, Ting Ma, Yanjun Fang

**Affiliations:** 1 The Key Laboratory of Risk Assessment and Control Technology for Environment and Food Safety, Tianjin Institute of Environment and Operational Medicine, Tianjin 300050, China; g2646391084@163.com (Y.G.); sunjran@163.com (J.S.); lmzazhu@163.com (M.L.); wujinlch@163.com (J.W.); quanbs@163.com (Z.Z.); luminary1004@163.com (T.M.); 2 Ningxia Hui Autonomous Region Food Testing Research Institute, Yinchuan 750000, China; 3 College of Chemistry and Chemical Engineering, Northwest Normal University, Lanzhou 730070, China

**Keywords:** Ratiometric fluorescence, 2,4-dichlorophenoxyacetic acid, Manganese dioxide, Carbon dots, Colorimetric detection

## Abstract

Nanomaterials are desirable for sensing applications. Therefore, MnO_2_ nanosheets and nitrogen-doped carbon dots (NCDs) were used to construct a ratiometric biosensor for quantification of 2,4-dichlorophenoxyacetic acid. The MnO_2_ nanosheets drove the oxidation of colorless o-phenylenediamine to OPDox, which exhibits fluorescence emission peaks at 556 nm. The fluorescence of OPDox was efficiently quenched and the NCDs were recovered as the ascorbic acid produced by the hydrolyzed alkaline phosphatase (ALP) substrate increased. Owing to the selective inhibition of ALP activity by 2,4-D and the inner filter effect, the fluorescence intensity of the NCDs at 430 nm was suppressed, whereas that at 556 nm was maintained. The fluorescence intensity ratio was used for quantitative detection. The linear equation was F = 0.138 + 3.863·C 2,4-D (correlation coefficient R^2^ = 0.9904), whereas the limits of detection (LOD) and quantification (LOQ) were 0.013 and 0.040 μg/mL. The method was successfully employed for the determination of 2,4-D in different vegetables with recoveries of 79%~105%. The fluorescent color change in the 2,4-D sensing system can also be captured by a smartphone to achieve colorimetric detection by homemade portable test kit.

## 1. Introduction

Food safety has emerged as a crucial human health issue owing to rapid developments in modern agricultural and food processing industries. Several serious food safety-related problems, such as food adulteration, contamination, and the incorporation of pesticide residues, have resulted in many poisoning events. 2,4-Dichlorophenoxyacetic acid (2,4-D) is extensively used in agricultural production as a pesticide and herbicide because of its remarkable effect on producing high yields by eradicating injurious insects [1]. Additionally, it is widely used as a fruit preservative to enhance fruit disease resistance, delay fruit senescence, and prolong fruit storage periods [2]. However, 2,4-D can progressively accumulate in environmental compartments, affect non-target organisms, and induce sublethal effects; these represent the most common environmental exposure scenarios [3].

A recent study [4] demonstrated that the ubiquity and high variability of urinary 2,4-D levels of pregnant women in central China across the three trimesters were associated with elevated exposure to 2,4-D. Moreover, the Infants’ Environmental Health study (ISA, Costa Rica) recommended implementing measures to reduce exposure to pesticides such as 2,4-D, which is believed to disturb thyroid function among pregnant women and even affect fetal and child development [5].

The insignificant degradation of 2,4-D in nature and its straightforward accumulation, causes considerable harm to human health and the ecological environment [6,7]. International organizations have established strict limits on 2,4-D pesticide residues in food crops, drinking water, and environmental standards [8]. Therefore, the development of rapid monitoring and detection methods for 2,4-D residues in routine life is crucial.

Traditional instrumental and biochemical methods, including enzyme-linked immunosorbent assay (ELISA) [9], microfluidics paper-based analytical devices (µ-PADs) [10], molecularly imprinted electrochemical sensing [11], and high-performance liquid chromatography (HPLC) [12], have been extensively employed for 2,4-D detection. To a certain extent, these techniques are effective and provide high sensitivity, selectivity, and precision for many severe detection-related scenarios. However, the widespread application of these methods has been limited because of their requirements for expensive instruments and skilled operators. Therefore, diverse alternative biosensors have been developed to achieve cost-effective, portable, simple, and real-time monitoring and detection of pesticides [13]. Although popular biosensing methods based on specific antigen–antibody binding exhibit good selectivity and high sensitivity, the preprocessing of antibodies is complex and the experimental environment is harsh [14]. Therefore, biosensors based on enzymes such as acetylcholinesterase (AchE) [15], horseradish peroxidase (HRP) [16] and alkaline phosphatase (ALP) [17] have been successfully used to analyze pesticides. The pesticide-induced specific inhibition of enzyme activity reduces the amounts of substances produced by enzymatic hydrolysis, thus affecting the pesticide residue detecting function of the probe or sensor.

Quantum dots have been successfully applied in drug delivery, biosensing, cell labeling, and imaging [18,19] because of their outstanding properties, such as photoluminescence, tunable spectra, and straightforward modification [20,21,22]. Nanosized fluorescent sensors [23] based on carbon quantum dots (CQDs) have garnered extensive attention owing to their noteworthy advantages, such as straightforward operation, rapid response, and adequate sensitivity. Manganese dioxide (MnO_2_) nanosheets are a two-dimensional nanomaterial with a large specific surface area, adequate catalytic oxidation performance, and wide absorption bands [24]. MnO_2_ nanosheets have been combined with scopoletin (SC) and Amplex Red (AR) to detect organophosphorus pesticides owing to their peroxidase-like catalytic oxidative properties [25]. Furthermore, the combination of CQDs and MnO_2_ nanocomposites [26] can significantly enhance pesticide [27] detection sensitivity and capacity.

Ratiometric fluorescent sensors [28] exhibit a considerably improved dynamic response range through changes in the intensity ratio and internal standard, which limit external interference [29,30]. Satisfactory results have been achieved using CdSe QDs as fluorescent sensors for 2,4-D detection [31]. However, the introduced heavy metal was less green and had potential risks. The carbon quantum dots were generally synthesized by the one-pot method using low toxicity raw materials [23].

**Scheme 1:**
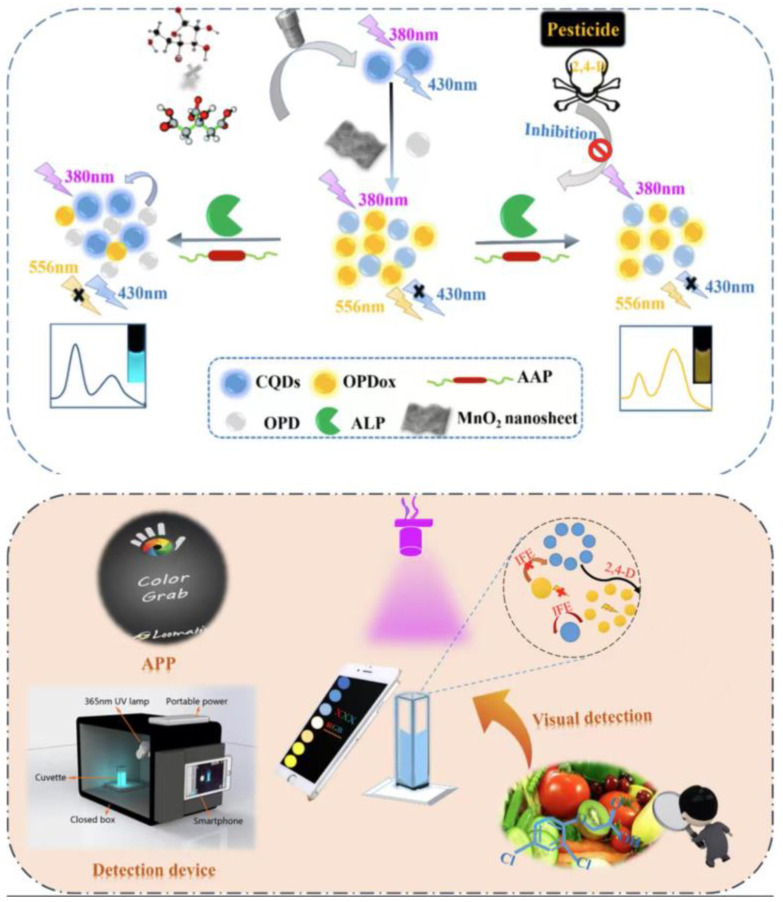
Illustration of the 2,4-D detection mechanism (**top**) and the strategy behind fluorescence-based optical sensing detection of 2,4-D (**bottom**).

In this study, a novel ratiometric fluorescent sensor was developed by combining MnO_2_ nanosheets and nitrogen-doped carbon dots (NCDs) for high-sensitivity analysis of 2,4-D. Owing to their catalytic oxidation activity, MnO_2_ nanosheets can oxidize o-phenylenediamine (OPD) into OPDox with yellow fluorescence. The blue fluorescence of the NCDs is quenched owing to the inner filter effect (IFE). The MnO_2_ nanosheets are reduced to Mn^2+^, and the formation of OPDox is consequently hindered by ascorbic acid (AA), which is produced via the ALP-driven hydrolysis of L-ascorbic acid 2-phosphate (AAP). The fluorescence signal of OPDox decreases, and that of the NCDs subsequently increases. In the presence of 2,4-D, ALP activity is inhibited during the reduction of the MnO_2_ nanosheets to Mn^2+^. Therefore, the fluorescence intensity of OPDox increases, whereas that of the NCDs decreases; these characteristics were leveraged to construct the sensitive ratiometric fluorescent sensor for 2,4-D or ALP detection (Scheme 1 top). Additionally, because the MnO_2_ nanosheet-driven oxidation of OPD exhibits a significant UV—vis absorption variation at 420 nm, 2,4-D can be detected using a standard UV—vis spectrophotometer. Moreover, fluorescence-based optical sensing detection can be realized by capturing the sharp color change in fluorescence using a smartphone. The designed sensing system, which is simple, highly sensitive, selective, and portable, can achieve a triple-detection mode including fluorescence spectrophotometer, ultraviolet-visible spectrophotometer and smartphone colorimetric devices for 2,4-D analysis.

## 2. Materials and Methods

### 2.1. Chemicals and Materials

Manganese chloride (MnCl_2_), hydrogen peroxide (H_2_O_2_), tetramethylammonium hydroxide (TMAH), sodium citrate, polyacrylamide, and OPD were provided by Sigma-Aldrich Co., Ltd. (Shanghai, China). 2,4-D and the other reagents used in the interference experiments were obtained from Aladdin Co., Ltd. (Shanghai, China). ALP (activity: 60,000 mU/mL) and AAP were purchased from Solarbio Science & Technology Co., Ltd. (Beijing, China). Ultrapure water (18.2 MΩ cm) was prepared using a Millipore water purification system. Unless otherwise stated, all the reagents were of AR grade.

### 2.2. Preparation of MnO_2_ Nanosheets

The MnO_2_ nanosheets were synthesized according to a previously reported protocol [32]. MnCl_2_ (0.3 M, 10 mL) was quickly added to a mixture of H_2_O_2_ (3%, 20 mL) and TMAH (0.6 M). The mixture was then stirred at room temperature for 12 h. The resulting black–brown solution was centrifuged (2000 rpm) and then washed three times with ultrapure water and methanol. The resulting precipitate, which represented bulk MnO_2_ nanosheets, was dried at 60 °C, dispersed in ultrapure water (1 mg/mL), and then sonicated for 10 h. The resulting solution was stored at 4 °C for subsequent experiments.

### 2.3. Preparation of NCDs

The NCDs were synthesized using a previously reported method [33]. Sodium citrate (1.0 g) and polyacrylamide (0.52 g) were dissolved in ultrapure water (20 mL). The resulting solution was vigorously stirred, transferred to a polytetrafluoroethylene-based high-pressure reactor (50 mL), and reacted at 200 °C for 3 h. After cooling to room temperature, the NCD solution was purified by 1000 ka dialysis for 12 h and stored at 4 °C for subsequent experiments.

### 2.4. Preparation of Standard Solution of 2,4-D

2,4-D (1mg/mL) was purchased directly with a CAS number of 94-75-7. Take 1mg/mL 2,4-D standard solution 1mL to volumetric flask, methanol fixed to 10mL, prepared as 100 μg/mL standard solution; respectively, take 100 μg/mL standard solution 100 μL, 200 μL, 250 μL, 500 μL, with a matrix blank solution to 1 mL, the preparation of 10 μg/mL, 20 μg/mL, 25 μg/mL, 50 μg/mL concentration of the series of standard solutions; and then 100 μL of each of the above standard solutions were taken up to form the standard solutions with concentrations of 0.05 μg/mL, 1 μg/mL, 2 μg/mL and 5 μg/mL.

### 2.5. Measurements of 2,4-D

A standard fluorescence spectrometer was used to detect 2,4-D. To this end, different concentrations of 2,4-D were added to a phosphate buffer solution (0.1 mM, pH 8) containing ALP (20 mU/mL). A phosphate buffer pH = 8 or pH = 5.5 was adjusted by 6 M NaOH and 6 M HCl solutions. The resulting mixture was then incubated at 37 °C for 10 min. AAP (3 mM, 100 μL) and an MnO_2_ nanosheet solution (0.2 mg/mL, 200 μL) were subsequently added to the mixture, and the resulting solution was incubated again for 30 min. Finally, OPD (10 mM, 150 μL) in phosphate buffer (pH 5.5) and the NCDs (40 μL) were introduced to the solution and reacted for 5 min. Finally, fluorescence measurements of the resulting solution were performed using fluorescence colorimetric apparatus, which has been comprehensively described in our previous article [21].

### 2.6. Sample Pretreatment

Different types of vegetables were sourced from local supermarkets in Lanzhou (China). Each sample (15 g) was placed in a centrifuge tube (100 mL) and homogenized by adding acetonitrile (30 mL). The tube was then centrifuged at 4000 rpm for 5 min. The supernatant (10 mL) was added to hydrochloric acid (1mol/L, 1.0 mL), followed by the addition of dried sodium chloride (2.5 g). The tube was then agitated, and the supernatant was concentrated using a vacuum rotary evaporator at 50 °C until it completely dried. The product was finally resolubilized with a methanol solution.

## 3. Results

### 3.1. Characterization of NCDs and MnO_2_ Nanosheets

**Figure 1:**
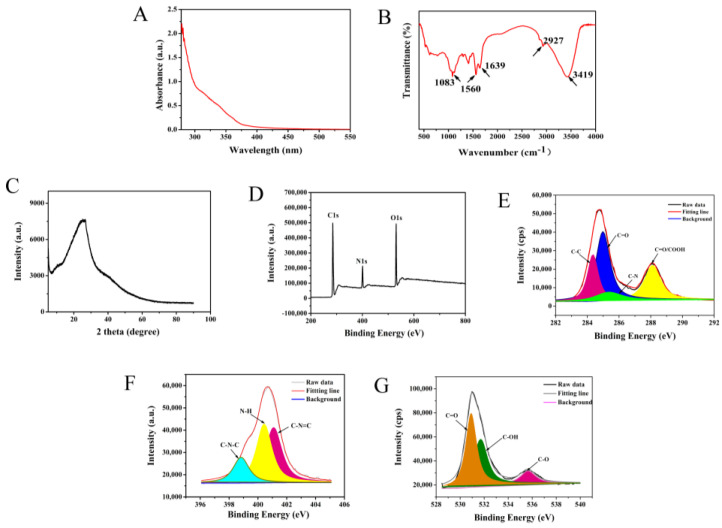
(**A**) UV-vis absorption spectrum, (**B**) FTIR spectrum, (**C**) XRD pattern, and (**D**) XPS profile of blue-emitting nitrogen-doped carbon dots (NCDs). High-resolution (**E**) C 1s, (**F**) N 1s, and (**G**) O 1s XPS profiles.

Transmission electron microscopy (TEM) images of the NCDs (Figure S1) revealed their good monodispersity in water and their diameter (10 nm). The shoulder peak at 260–290 nm in the UV-vis absorption spectrum (Figure 1A) was probably caused by aromatic groups on the surface of the NCDs; Fourier-transform infrared spectroscopy (FTIR) analysis (Figure 1B) indicated that the NCDs exhibited a characteristic band at 3419 cm^−1^, which was assigned to an N-H stretching vibration. The additional characteristic peaks at 1639, 1560, and 1083 cm^−1^ represent the stretching vibrations of C=O, C=C, and C-N, respectively. X-ray diffraction (XRD) analysis of the NCDs (Figure 1C) revealed a broad peak at a 2θ value of 19.98°, which was attributed to disordered carbon atoms and the graphite lattice spacing. X-ray photoelectron spectroscopy (XPS) analysis of the NCDs (Figure 1D) revealed characteristic peaks at 284.07, 402.44, and 530.40 eV, corresponding to C1s, N1s, and O1s, respectively. In combination with the FTIR data, these results confirm the presence of numerous carboxyl and ammonia groups on the surface of the NCDs. The C1s peak was deconvoluted into four peaks at 284.33, 284.97, 285.45 and 288.19 eV, which corresponded to the C-C, C-O, C-N, and C=O/COOH bonds, respectively (Figure 1E). The deconvolution of the O1s peak yielded three peaks at 530.96, 531.72, and 535.60 eV (Figure 1F), which were attributed to C=O, C-OH, and C-O, respectively.

**Figure 2:**
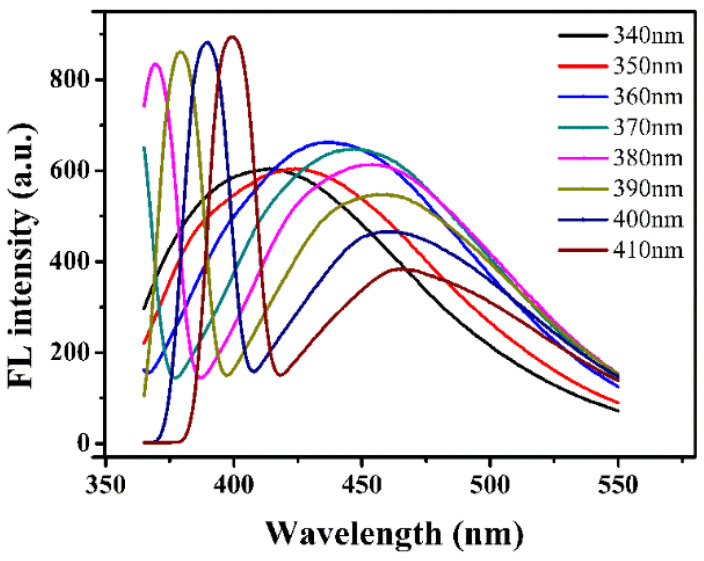
Fluorescence emission of nitrogen doped blue carbon dots (NCDs) at different excitation wavelengths.

**Figure 3:**
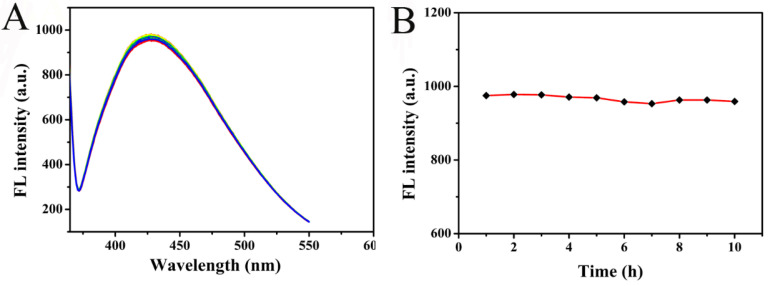
(**A**) Fluorescence spectra of nitrogen-doped carbon dots and (**B**) fluorescence intensity changes with time.

The high-resolution N1s spectrum (Figure 1G) comprised three peaks at 398.79, 400.44, and 401.08 eV, which were ascribed to the C-N-C, N-H, and C-N=C bonds, respectively. Adjustment of the excitation wavelength from 290 to 400 nm (Figure 2) led to a rightward shift in the fluorescence emission peak, indicating that the NCDs had wavelength-dependent excitation. The fluorescence excitation wavelength was determined to be 350 nm, and the maximum emission peak appeared at 430 nm. The blue fluorescence of the NCDs was stable for 10 h (Figure 3A,B), which highlighted the good resistance of the NCDs to photobleaching.

The MnO_2_ nanosheets appeared as a typical two-dimensional fabric with several folds and crinkles (Figure S2A). The two-dimensional nanosheets exhibited a wide UV-vis absorption band from 300 to 700 nm, with a maximum absorbance peak at 380 nm (Figure S2B). The characteristic peak, centered at 520 nm in the FTIR spectrum (Figure S2C), resulted from the Mn-O bond, which confirmed the synthesis of the MnO_2_ nanosheets. XRD analysis (Figure S2D) revealed characteristic diffraction peaks at 21.9°, 37.2°, 42.5°, 56°, and 67.3°, which were attributed to the (001), (002), (100), and (110) crystal planes of the MnO_2_ nanosheets, respectively. The strong signals at 289.08, 406.32, 538.27, and 640.91 eV in the XPS profile (Figure S2E) validated the presence of Mn, O, N, and C elements in the prepared nanomaterial.

### 3.2. Principle of the NCD-Based Ratiometric Fluorescence Sensing and Colorimetric Response

No significant fluorescence was observed when OPD or the MnO_2_ nanosheets were present individually (Figure S3). The MnO_2_ nanosheets exhibited decent catalytic oxidation activity. When the MnO_2_ nanosheets were reacted with OPD for a certain duration, the colorless and nonfluorescent OPD was oxidized into OPDox, which exhibited strong yellow fluorescence with an emission peak at 560 nm under 410 nm excitation. Moreover, OPDox exhibited a noticeable absorption peak at 420 nm. The distinct yellow color was visible to the naked eye.

The prepared NCDs exhibited intense blue fluorescence with an emission peak at 430 nm under 360 nm excitation. The absorption band of OPDox substantially overlapped with the emission peak of the NCDs (Figure S4A). When the NCDs were introduced to the MnO_2_–OPD system, the fluorescence of the NCDs was quenched by OPDox. Zeta potential analysis (Figure S4B) suggested that the NCDs and OPDox had potentials of 12 and 10 mV, respectively. Their positive charges indicated their aversion to electrostatic attraction, thereby highlighting the probable role of the IFE mechanism in the fluorescent response [34].

MnO_2_ nanosheets, which function as a recognizer, can be reduced to Mn^2+^ using reductants such as AA. The AA product that originates during the hydrolysis of ALP plays the same role in the oxidation–reduction process described above. ALP and the AAP substrate did not show distinct emission in the fluorescence spectra (Figure S5). When ALP-AAP was conjugated to the MnO_2_-OPD-NCD system, a significant variation in fluorescence intensity was observed. In the presence of a certain amount of AAP, ALP produced more AA owing to its higher activity, which generated a smaller amount of OPD because of the consumption of large quantities of the MnO_2_ nanosheets. The fluorescence of the blue NCDs recovered to higher intensity levels. However, integrating ALP or AAP separately with MnO_2_ had no effect on the oxidation of OPD.

As an enzyme inhibitor, 2,4-D can selectively inhibit ALP activity, which provides an effective basis for the development of a 2,4-D sensor. The distinct color change in fluorescence from blue to yellow, which was visible to the naked eye, could be captured by a smartphone for analyzing the stimulus response of ALP-MnO_2_-OPD toward 2,4-D. Similarly, the absorbance of the oxidized product—OPDox—gradually changed owing to the unique reducing behavior of AA (Figure S6). The UV—vis absorbance of OPDox at 420 nm showed a noticeable change upon the introduction of ALP and 2,4-D, which was accompanied by a color change from yellow to colorless. Scheme 1 (top) illustrates the 2,4-D detection mechanism exploited in this study. Colorimetric analysis was performed during the procedure described above.

### 3.3. Optimization of Conditions for Ratiometric Fluorescent Determination of 2,4-D

Various experimental parameters such as pH, incubation time, and substrate concentration were optimized. With respect to the pH-dependent changes in the fluorescence intensity at 556 nm, the highest emission intensity was achieved at pH 5.5 (Figure S7A). In terms of the incubation time (Figure S7B), the fluorescence intensity increased with the duration and then stabilized after 5 min. Therefore, an incubation time of 5 min was selected for the subsequent experiments. The amounts of the MnO_2_ nanosheets and OPD were particularly important for realizing target detection. The fluorescence intensity at 556 nm continuously increased with the addition of OPD from 0 to 10 mM and decreased at concentrations greater than 10 mM (Figure S7C). At the OPD concentration of 10 mM, the fluorescence intensity at 556 nm gradually increased with increasing MnO_2_ content. Therefore, an MnO_2_ solution concentration of 0.2 mg/mL was used in the subsequent experiments, with consideration of the visual detection and sensitivity aspects.

With the assistance of ALP, the AAP substrate can be hydrolyzed to produce AA. The conditions for hydrolysis were optimized at a temperature of 37 °C. In terms of the duration of hydrolysis, the fluorescence intensity initially decreased with time and stabilized after 30 min of enzymatic hydrolysis (Figure S7D). Similar results were obtained by analyzing the fluorescence quenching efficiency. Thus, a duration of 30 min was selected for achieving enzymatic hydrolysis. With respect to the ALP concentration, ALP with a small amount of AAP was unable to generate sufficient amounts of AA (Figure S7E), whereas an excessive amount of AAP required a large amount of ALP to produce AA at substantial levels. Therefore, 3 mM was selected as the AAP concentration for the enzymatic hydrolysis. For the enzyme inhibition, the ALP activity was fixed at 20 mU/mL and the inhibitory effect of 2,4-D was evaluated. As shown in Figure S7F, 2,4-D exerted the maximum effect, and the value of F_556_/F_430_ plateaued after 10 min, suggesting that 10 min was sufficient for AAP hydrolysis.

### 3.4. Analytical Performance

A quantitative analysis of 2,4-D was performed under the optimized conditions. To that end, a standard curve was constructed by plotting the fluorescence intensity ratio (F_556_/F_430_) against the 2,4-D concentration.

**Figure 4:**
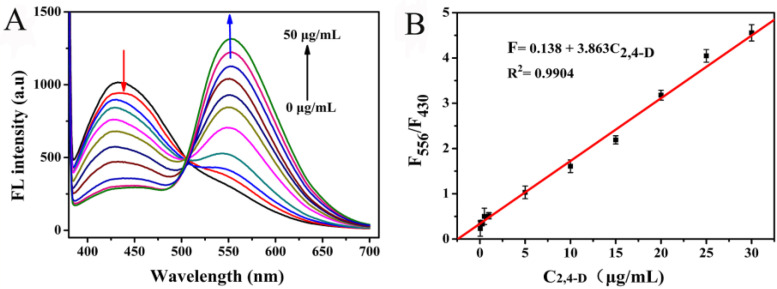
(**A**) Fluorescence spectra acquired for 2,4-D detection by ratiometric fluorescence sensing and (**B**) the linear relationship between F_566_/_430_ and 2,4-D concentration (*n* = 3).

The enzyme-inhibiting ability of 2,4-D gradually improved with increasing 2,4-D concentration (Figure 4A). The increase and decrease in the fluorescence intensity at 556 and 430 nm, respectively, enabled the ratiometric fluorescence-based determination of 2,4-D. Under the optimized conditions, a satisfactory linear relationship was obtained between the fluorescence intensity ratio (F_556_/F_430_) and concentrations from 0.05 to 30 μg/mL (Figure 4B). The linear equation was F = 0.138 + 3.863·C_2,4-D_ (correlation coefficient R^2^ = 0.9904), whereas the limits of detection (LOD) and quantification (LOQ) were 0.013 and 0.040 μg/mL. The limit of detection (LOD) was calculated based on the rule of 3σ/slope (σ is the standard deviation of the reagent blank). The limit of quantitation (LOQ) was calculated according to a 10σ/slope. The low detection limit suggests the high sensitivity of the designed sensing strategy. The analytical performance of the developed sensor is compared with that of previously reported sensors in Table S1 [20,35,36,37].

### 3.5. UV—Vis Detection of 2,4-D

**Figure 5:**
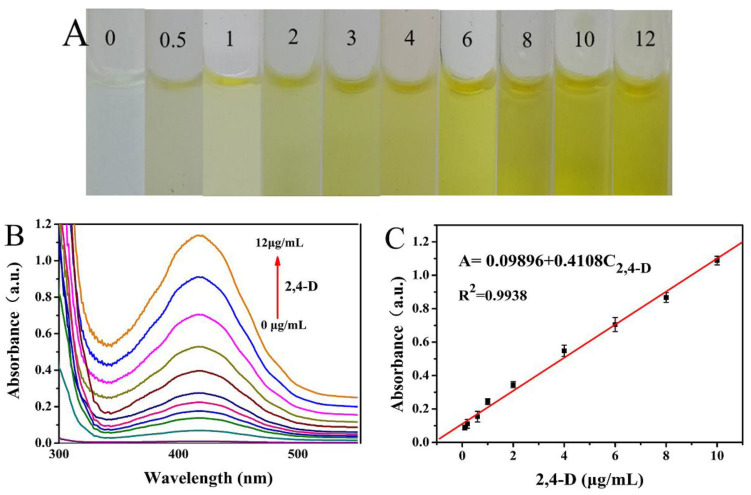
(**A**) Color changes of the OPDox observed in the presence of different concentrations of 2,4-D; (**B**) UV—vis spectra of 2,4-D at different concentrations, and (**C**) the linear relationship between the UV absorption intensity at 420 nm and 2,4-D concentration.

Because of the catalytic oxidative action of the MnO_2_ nanosheets and the enzyme-inhibiting tendency of 2,4-D, the solution of the oxidation product—OPDox—showed a gradually intensifying yellow color with the different concentrations of the 2,4-D (Figure 5A). The UV-vis absorbance at 420 nm (A420) increased with increasing 2,4-D concentration (Figure 5B). Moreover, an adequate linear relationship was obtained between A420 and 2,4-D concentrations between 0.5 and 10 μg/mL (Figure 5C). The linear equation was A420 = 0.09896 + 0.4108·C_2,4-D_ (R^2^ = 0.9938), whereas the LOD and LOQ were 0.18 and 0.53 μg/mL, respectively.

### 3.6. Fluorescence-Based Optical Sensing

**Figure 6:**
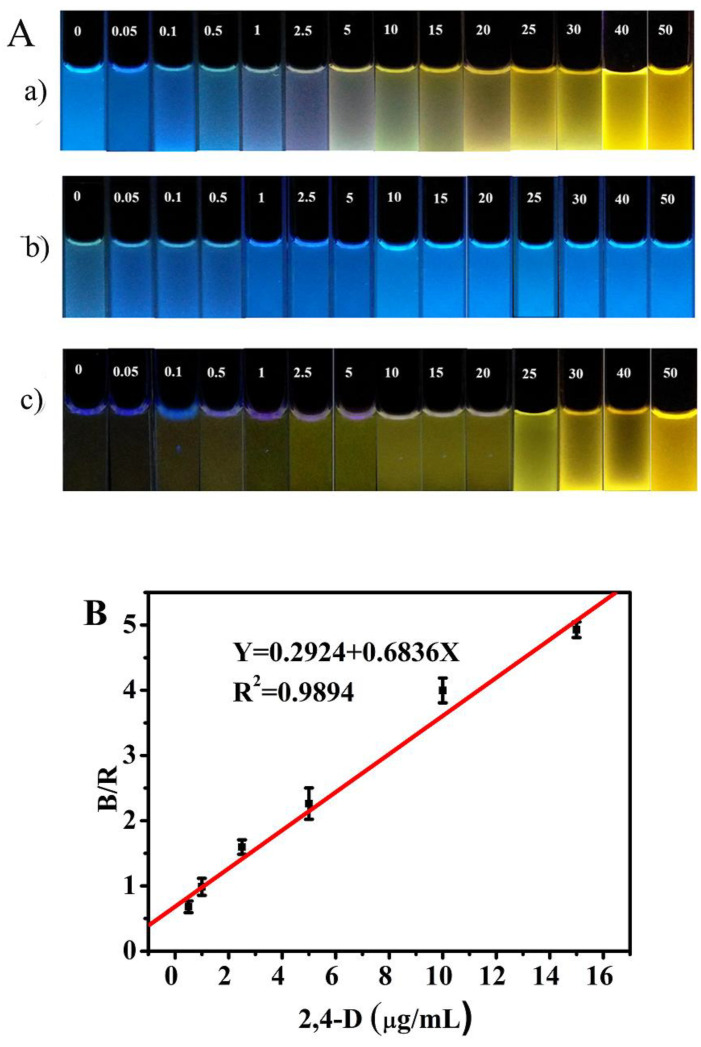
(**A**) Different fluorescence color changes of the 2,4-D detection with (**a**) ratiometric fluorescent sensor comprising MnO_2_ nanosheets and carbon dots, (**b**) single-emission fluorescent sensors containing carbon dots, and (**c**) o-phenylenediamine. (**B**) shows the relationship between B/R values and the 2,4-D concentration.

A controlled experiment was performed to demonstrate the superiority of the ratiometric fluorescence-based detection system (Scheme 1 bottom). MnO_2_-NCDs were first used to respond to 2,4-D without introducing OPD. As shown in Figure 6A(b), no significant fluorescence-related color changes were observed when only the NCDs were used for 2,4-D detection. During the operation of the NCD-free single-emission sensor containing MnO_2_ and OPD, ALP started disintegrating the MnO_2_ nanosheets, which prevented the generation of OPDox. The fluorescence color became dim, dark, and sometimes barely discernible (Figure 6A(c)). However, the distinct shift in fluorescence color from blue to yellow (Figure 6A(a)) can provide insight into the tracking target using a homemade smartphone-based colorimetric device [21] coupled with the Android application Color Grab [38]. To eliminate the systematic errors generated during these measurements, the following parameters associated with the smartphone-based platform were optimized: the light source, dark box material, position of the light source, and distance between the light source and cuvette.

After directly extracting the RGB values from the images of the solution obtained using a 365 nm UV lamp, diverse mathematical models were established for quantitative analysis. The B/R parameter was selected to determine the ratio of the B and R channels. A calibration curve was obtained for 2,4-D concentrations of 1.0–15 μg/mL (Figure 6B). The corresponding linear profile can be expressed as Y = 0.292 + 0.684X (regression coefficient = 0.9894), whereas its LOD and LOQ were 0.42 and 0.95 μg/mL, respectively. Although the detection limit obtained using the smartphone-based strategy is not as low as that determined using the fluorimeter, the visual and quick readout aspects of the former emphasize the strength of such portable and low-cost devices for use in on-site testing.

### 3.7. Comparison of the Devised Strategies

The three detection strategies reported herein were compared based on the aforementioned results. The single-emission fluorescence detection scheme showed a relatively narrow linear range and slightly higher detection limits, with the LOD and LOQ being 0.18 and 0.53 μg/mL, respectively, whereas the dual-emission ratiometric fluorescence strategy exhibited higher sensitivity (LOD and LOQ were 0.013 and 0.040 μg/mL, respectively). The LOD and LOQ of the fluorescence-based optical sensing methods were 0.42 and 0.95 μg/mL, respectively. The sensitivities of the UV-based and smartphone-assisted colorimetric methods were lower than that of direct fluorescence determination; however, the results obtained through the UV- and smartphone-based detection strategies were essentially identical. This is because the smartphone-based colorimetric detection and UV—vis methods are complementary. The UV—vis method can effectively prevent the interference of external conditions such as temperature during the determination, whereas the smartphone-based colorimetric method offers straightforward operation, portability, visualization, detection, and field deployment ability.

### 3.8. Selectivity and Anti-Interference Capability

The selectivity of the enzyme-triggered ratiometric fluorescence system was evaluated using various interfering substances under the same experimental conditions. The interfering species included common cations (Na^+^, K^+^, Mg^2+^, Ca^2+^, Al^3+^, Zn^2+^, Fe^3+^, Pb^2+^, Hg^2+^, Co^2+^, Ni^2+^, Cu^2+^, and Cd^2+^); anions (Cl^−^, SO_4_^2−^, and PO_4_^3−^); and pesticides (fipronil, dithianon, fenvalerate, methomyl, paraoxon, pirimicarb, isoprocarb, parathion-methyl, 1-naphthylacetic acid, and propoxur). The tolerance limit of different substances is defined as the largest amount making a variation of less than ±5% in the determination of 2,4-D with a concentration of 0.5 μg/mL. The tolerance limits of the various interfering species are listed in Table 1. The results indicate that the ratiometric fluorescence sensing system demonstrated superior selectivity toward 2,4-D.

### 3.9. Determination of 2,4-D in Real Samples

The constructed ratiometric fluorescent sensor was used to detect 2,4-D in the following real samples to verify its validity and practicability: cauliflower, cucumber, cabbage, celery, spinach, and bean sprouts. The resulting data were compared with those obtained by HPLC analysis. The real samples were spiked with different concentrations of 2,4-D (0.05, 1, and 4 μg/mL), which resulted in recovery rates of 79% to 105% (Table 2). The t-values obtained according to the *t*-test were less than 2.78, indicating the lack of statistically significant differences between the ratiometric fluorescence-based approach and the HPLC method. The acceptability of these results indicates that sensitive detection of trace amounts of 2,4-D in real samples can be achieved using the designed ratiometric fluorescent system.

## 4. Conclusions

The detection of 2,4-D in various samples was realized using a ratiometric fluorescent sensor that comprised MnO_2_ nanosheets and NCDs. The platform exhibited high sensitivity and selectivity, with the latter ascribed to the specific recognition of 2,4-D by ALP. Additionally, colorimetric detection of 2,4-D was performed using a UV—vis spectrophotometer and a smartphone. The substantial change in the fluorescence color combined with the smartphone-based detection enabled visual quantitative analysis of the target. The devised system is portable, cost-effective, and can achieve rapid on-site detection of pesticide residues in food samples.

## Figures and Tables

**Table 1:** Tolerance ratios for the determination of 2,4-D (0.5 μg/mL).

Interfering Substances	Tolerance Limit
K^+^, Na^+^, Ca^2+^, Co^2+^, Ba^2+^, Zn^2+^, Al^3+^ Mg^2+^, and Cd^2+^	>1000
Cl^−^, SO_4_^2−^, and PO_4_^3−^	>1000
Fe^3+^, Pb^2+^, and Hg^2+^	600
Fipronil and dithianon	500
Fenvalerate Methomyl, paraoxon, and pirimicarb	120 350
Isoprocarb and parathion-methyl	200
1-Naphthylacetic acid	60
Propoxur	220

**Table 2:** Results of the determination of 2,4-D in various vegetables.

Sample	Spiked Concentration (μg/mL)	Concentration Estimated by HPLC (μg/mL)	Detected ^a^ (μg/mL)	Recovery (%)	t-Value ^b^
2,4-D (50 μg/mL ^c^)	0	50.8	49.2	/	0.72
Cucumber	0	0.54	0.52 ± 0.02	/	0.54
	0.05	/	0.57 ± 0.02	80	/
	1	/	1.46 ± 0.04	94	/
	4	/	4.58 ± 0.02	101	/
Cauliflower	0	/	/	/	/
	0.05	0.06	0.04 ± 0.01	80	1.46
	1	/	0.98 ± 0.02	98	/
	4	/	4.27 ± 0.04	107	/
Bean sprouts	0	/	/	/	/
	0.05	0.07	0.05 ± 0.02	89	2.32
	1	/	1.09 ± 0.03	109	/
	4	/	3.86 ± 0.01	97	/
Celery	0	/	/	/	/
	0.05	0.06	0.04 ± 0.02	79	1.61
	1	/	0.88 ± 0.03	87	/
	4	/	3.98 ± 0.06	100	/
Cabbage	0	/	/	/	/
	0.05	0.06	0.05 ± 0.01	98	1.12
	1	/	1.14 ± 0.04	112	/
	4	/	3.95 ± 0.04	98	/
Spinach	0	/	/	/	/
	0.05	0.05	0.04 ± 0.01	84	0.96
	1	/	0.92 ± 0.02	93	/
	4	/	4.10 ± 0.04	103	/

^a^ Mean value ± standard deviation (SD., n = 5) and 95% confidence limit. ^b^ Tabulated t-Value three degrees of freedom at 95% confidence level is 2.78. ^c^ 50 μg/mL is the concentration of 2,4-D standard samples.

## Data Availability

The data presented in this study are available on request from the corresponding authors.

## Supplementary Materials

The following supporting information can be downloaded at https://www.mdpi.com/article/10.3390/bios14020063/s1, Figure S1. (A) TEM image. Figure S2. (A) TEM image of manganese dioxide nanosheets (MnO_¬2_), (B) UV-Vis absorption spectrum of MnO_¬2_, (C) FT-IR spectrum of MnO_¬2_, (D) XRD pattern of MnO_2_ and (E) XPS survey spectrum of MnO_2_. Figure S3. Fluorescence spectra of various substances (a) MnO_2_, (b) OPD, (c) MnO_2_+OPD, (d) NCDs, (e) MnO_2_+NCDs, (f) MnO_2_+OPD+NCDs. Figure S4. (A) Uv-vis absorption spectrum of OPDox and fluorescence emission spectrum of NCDs (EX=360nm), (B) the potential diagram of OPDox and NCDs. Figure S5. Fluorescence spectra of ratio fluorescence system before and after adding ALP and 2, 4-D, before: (a) MnO_2_+OPD+NCDs, after: (b) MnO_2_+OPD+NCDs +ALP+AAP, (c)MnO_2_+OPD+NCDs+ALP+AAP+2,4-D, (d) MnO_2_+OPD+NCDs +2,4-D, (e) MnO_2_+OPD+NCDs+AAP, (f) MnO_2_+OPD+NCDs+ALP. Figure S6. Uv-vis absorption spectra of various substances (a) OPD, (b) ALP, (c) AAP, (d) ALP+AAP, (e) MnO_2_, (f) MnO_2_+OPD+AAP+ALP, (g) MnO_2_+OPD+AAP+ALP+2,4-D, (h) MnO_2_+OPD+2,4-D. Figure S7. Effects of (A) OPD-oxidation-related pH, (B) oxidation time, (C) concentrations of OPD and the MnO_2_ nanosheets, (D) ALP hydrolysis time, (E) concentration of the hydrolyzed AAP substrate, and (F) time required for enzyme inhibition by 2,4-D. Table S1. Comparison of the 2,4-D sensor reported herein with similar previously reported sensors.
