# Supplementary material for: A Ratiometric Biosensor Containing Manganese Dioxide Nanosheets and Nitrogen-Doped Quantum Dots for 2,4-Dichlorophenoxyacetic Acid Monitoring
Source: Biosensors (Basel). 2024 Jan 24;14(2):63. doi: 10.3390/bios14020063 (PMC10887317; doi:10.3390/bios14020063)
Supplement: Supplementary file 1 [file biosensors-14-00063-s001.zip › biosensors-2807614-supplementary.pdf]

Electronic Supporting Material (ESM)

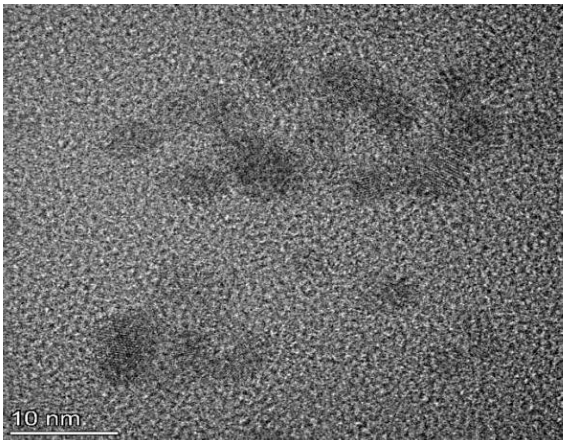

Figure. S1 (A) TEM image,

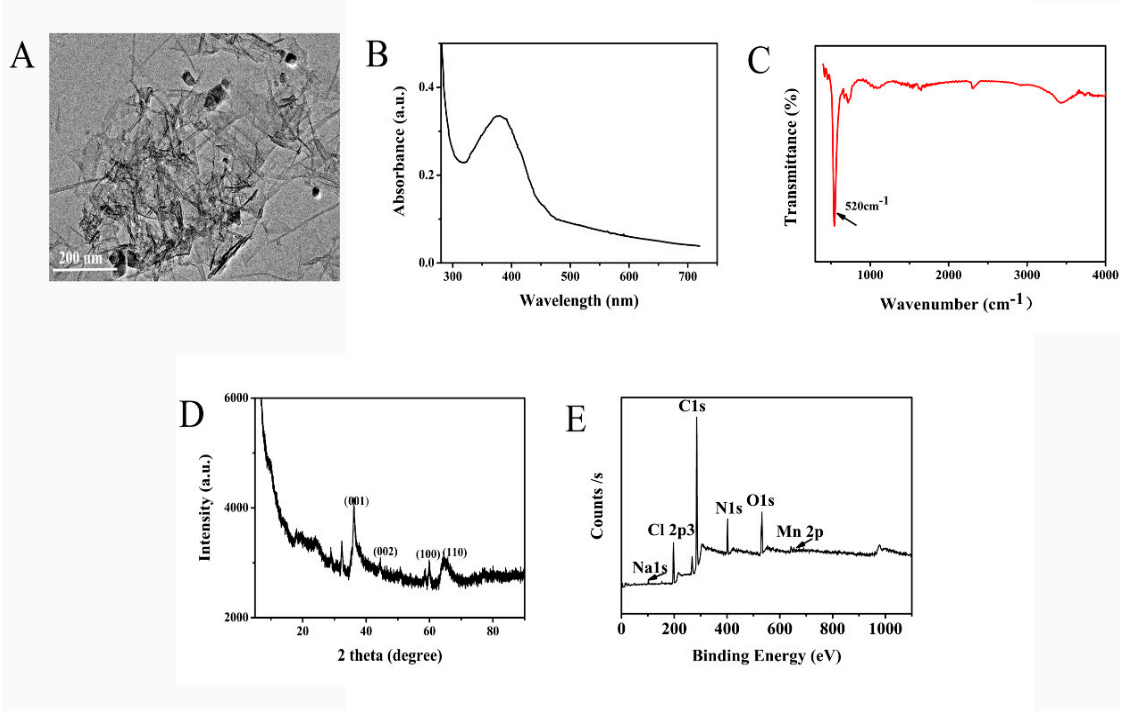

Figure.S2 (A) TEM image of manganese dioxide nanosheets (MnO_2), (B) UV-Vis absorption spectrum of MnO_2, (C) FT-IR spectrum of MnO_2, (D) XRD pattern of MnO_2 and (E) XPS survey spectrum of MnO_2.

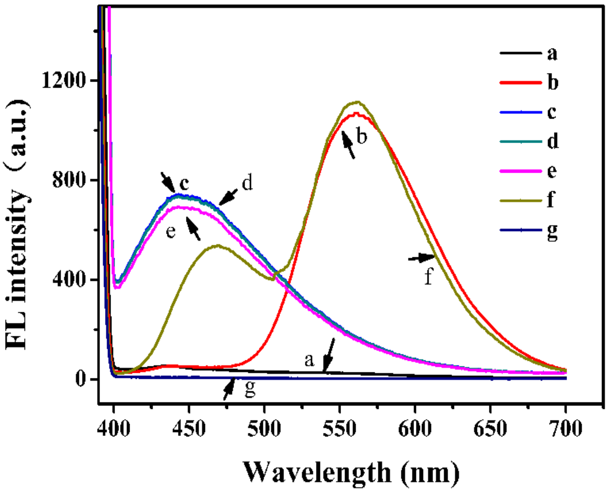

Figure.S3 Fluorescence spectra of various substances (a) MnO_2, (b) OPD, (c) MnO_2+OPD, (d) NCDs, (e) MnO_2+NCDs, (f) MnO_2+OPD+NCDs

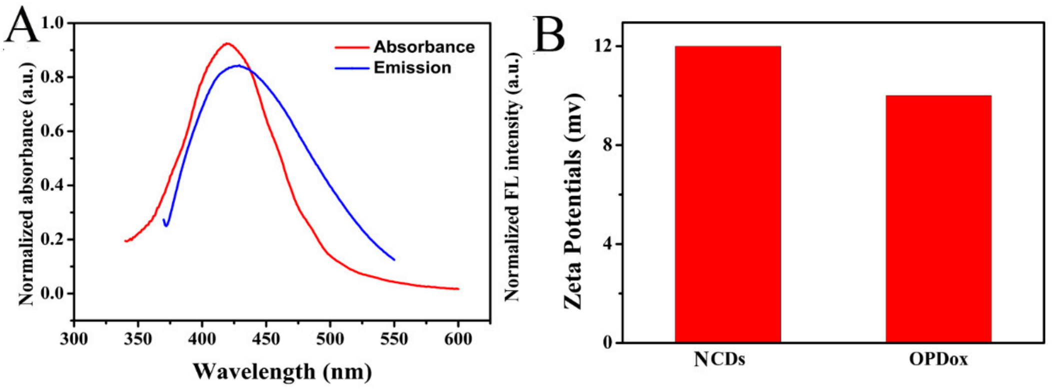

Figure.S4 (A) Uv-vis absorption spectrum of OPDox and fluorescence emission spectrum of NCDs（EX=360nm）， (B) the potential diagram of OPDox and NCDs

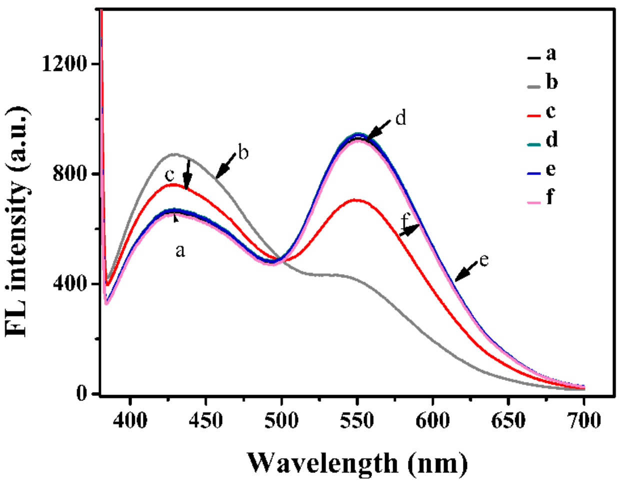

Figure.S5 Fluorescence spectra of ratio fluorescence system before and after adding ALP and 2, 4-D, before：(a) MnO_2+OPD+NCDs，after：(b) MnO_2+OPD+NCDs +ALP+AAP，(c) MnO_2+OPD+NCDs+ALP+AAP+2,4-D，(d) MnO_2+OPD+NCDs +2,4-D, (e) MnO_2+OPD+NCDs+AAP，(f) MnO_2+OPD+NCDs+ALP.

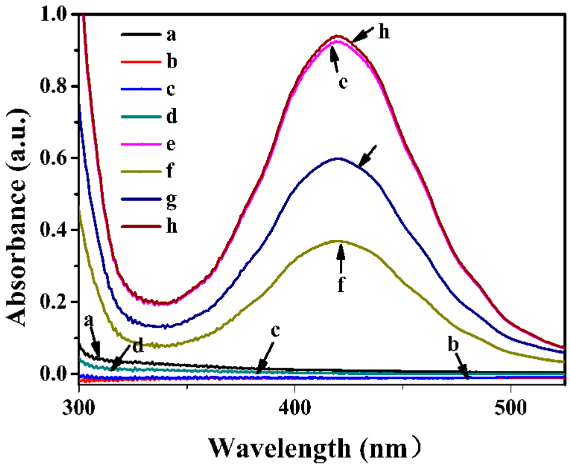

Figure. S6 Uv-vis absorption spectra of various substances (a) OPD, (b) ALP, (c) AAP, (d) ALP+AAP, (e) MnO_2, (f) MnO_2+OPD+AAP+ALP, (g) MnO_2+OPD+AAP+ALP+2,4-D, (h) MnO_2+OPD+2,4-D

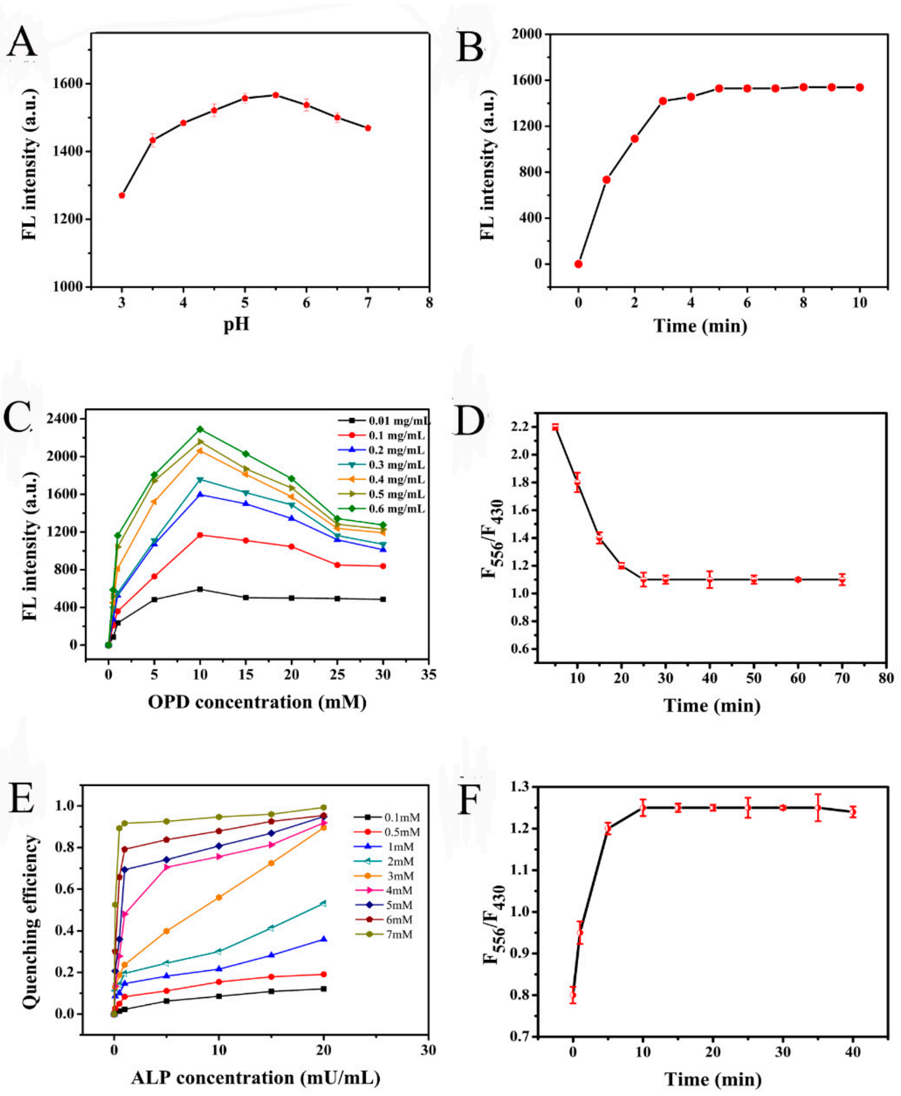

Figure.S7 Effects of (A) OPD-oxidation-related pH, (B) oxidation time, (C) concentrations of OPD and the MnO_2 nanosheets, (D) ALP hydrolysis time, (E) concentration of the hydrolyzed AAP substrate, and (F) time required for enzyme inhibition by 2,4-D

Table S1 Comparison of the 2,4-D sensor reported herein with similar previously reported sensors

Method	Linear range	Detection limit	Type	Reference
Electrochemistry	100–1000 nM	34 nM	Boron-doped diamond electrode	(Neto ,Oliveira,&,Suarez,2022)

Fluorescence	0–5 μg L^{-1}	50 ng mL^{-1}	Gold nanobipyramids sensors	(Ye, Zhang,& Wang, 2022)
Electrochemistry	0.1–1 mg L^{-1}	50 μg L^{-1}	Prussian blue nanoparticles	(Arduini, Moscone, 2019)
Electrochemistry	1.4–2.7 μM	0.21μM	Fe_3O_4-polyaniline nanocomposite	(Goswami ,Mahanta, 2021)
Ratiometric fluorescence	0.05–30 μg/mL	0.013 μg/mL	MnO_2-NCDs-OPD	Present study

References

Neto J C D, Dos Santos V B, de Oliveira S C B, Suarez W T, de Oliveira J L (2022) In situ voltammetric analysis of 2, 4-dichlorophenoxyacetic acid in environmental water using a boron doped diamond electrode and an adapted unmanned air vehicle sampling platform. Anal. Methods 14(13):1311-1319

Ye X, F, Yang L, Yang W, Zhang L, Wang Z (2022) Paper-based multicolor sensor for on-site quantitative detection of 2,4-dichlorophenoxyacetic acid based on alkaline phosphatase-mediated gold nanobipyramids growth and colorimeter-assisted method for quantifying color. Talanta 245:123489

Arduini F, Cinti S, Caratelli V, Amendola L, Palleschi G, Moscone D (2019) Origami multiple paper-based electrochemical biosensors for pesticide detection. Biosens. Bioelectron. 126:346-354

Goswami B, Mahanta D (2021) Fe3O4-Polyaniline Nanocomposite for Non-enzymatic Electrochemical Detection of 2,4-Dichlorophenoxyacetic Acid. ACS Omega 6(27):17239-17246
